# Co-expression Network Revealed Roles of RNA m^6^A Methylation in Human β-Cell of Type 2 Diabetes Mellitus

**DOI:** 10.3389/fcell.2021.651142

**Published:** 2021-05-18

**Authors:** Cong Chen, Qing Xiang, Weilin Liu, Shengxiang Liang, Minguang Yang, Jing Tao

**Affiliations:** The Institute of Rehabilitation Industry, Fujian University of Traditional Chinese Medicine, Fuzhou, China

**Keywords:** RNA m^6^A methylation, type 2 diabetes mellitus, co-expression network, RNA m^6^A methyltransferase, insulin

## Abstract

RNA m^6^A methylation plays an important role in the pathogenesis of type 2 diabetes mellitus (T2DM). RNA modifications and RNA-modifying regulators have recently emerged as critical factors involved in β-cell function and insulin resistance, including “writers,” “erasers,” and “readers.” However, their key roles in regulating gene expression in T2DM remain unclear. The construction of co-expression network could provide a cue to resolve this complex regulatory pathway. We collected the transcriptome datasets of β-cell in diabetic patients, calculated the partial correlation coefficient, excluded the influence from control variables of diabetes related genes, and identified the genes significantly co-expressed with m^6^A regulators. A total of 985 genes co-expressed with m^6^A regulators (Co-m^6^AR) were identified, which were enriched in metabolic process, MAPK and EGFR signaling pathways. Some of them have been confirmed to play a pivotal role in T2DM, including *CCNL2*, *CSAD*, *COX5A*, *GAB2*, and *MIRLET7I*, etc. Further, we analyzed the m^6^A modification characteristics of Co-m^6^AR in β-cell and identified 228 Co-m^6^AR containing m^6^A methylation sites, involving in several key signaling pathways regulating T2DM. We finally screened out 13 eQTL-SNPs localized in Co-m^6^ARs, and 4 have been reported strongly associated with diabetes, including *GAB2*, *LMNB2*, *XAB2*, and *RBM39*. This co-expression analysis provides important information to reveal the potential regulatory mechanism of RNA m^6^A methylation in T2DM.

## Introduction

Type 2 diabetes mellitus (T2DM) is considered a major health problem worldwide. This complicated metabolic disorder is characterized by chronic hyperglycemia, which can trigger β-cell function impairment, insulin resistance, and deficiency. However, the pathogenesis of T2DM remains unclear. The critical factor of T2DM is an inadequate functional islet β-cell mass, and a variety of risk factors can lead to islet β-cell dysfunction, either due to a reduced β-cell mass, or to inadequate β-cell phenotype and functional maturity (Dayeh and Ling, 2015). Insulin dysfunction can induce toxicity in different tissues leading to pathological and functional changes in multiple organs (Singh et al., 2001; Giacco and Brownlee, 2010; Reusch and Manson, 2017). A classical insulin resistance mechanism consists in dysregulated insulin signaling with impaired phosphorylated insulin receptor substrates (IRSs)(Copps et al., 2016) and other altered factors such as insulin-regulated Ser/Thr kinases, AMP-activated protein kinase, and glucose transporter 4.

Epigenetic modifications on DNA, RNA, and proteins may provide the link gene expression into pathological mechanisms of T2DM. Studies have shown different patterns of epigenetic regulation of DNA and proteins, but RNA modifications in T2DM remain poorly understood (He, 2010). N6-methyladenosine (m^6^A) is one of the most abundant post-transcriptional modifications on eukaryote RNA. Over 7000 human transcripts contain at least one m^6^A site, enriched in the coding sequence and the 3’untranslated region (3’UTR), near the stop codon of mature polyadenylated mRNAs (Dominissini et al., 2012; Meyer et al., 2012). RNA m^6^A modifications of the eukaryotic transcriptome can modulate mRNA splicing, export, localization, translation, and stability (Wang et al., 2014; Liu et al., 2015). The function of this modification is controlled by a series m^6^A regulator. Intracellular m^6^A regulation is dynamic and reversible, which is composed of methyltransferases (writers) and demethylases (erasers), including *METTL3*, *METTL14*, *WTAP*, *KIAA1429*, and *RBM15* (Bokar et al., 1997; Liu et al., 2014), fat mass and obesity-associated protein (*FTO*) (Jia et al., 2011) and alkylation repair homolog protein 5 (*ALKBH5*) (Zheng et al., 2013; Fedeles et al., 2015). On the other hand, recruiting m^6^A-binding proteins called “readers” can specifically bind to m^6^A-modified transcripts including the nuclear *YTHDC1* and the cytoplasmic *TYHDC2*, *YTHDF1*, *YTHDF2*, and *YTHDF3* (Wang et al., 2014).

Homeostasis of m^6^A modifications on RNA is essential for the regulation of human transcripts expression in T2DM. Demethylase *FTO* is the first gene strongly associated with adipose mass and obesity in β-cells (Scuteri et al., 2007; Taneera et al., 2018), positively correlated with serum glucose (Blauth and Falliner, 1986), and closely associated with metabolic alterations, cardiovascular diseases, and T2DM (Boissel et al., 2009; Church et al., 2010; Lee et al., 2014). *FTO* knockout mice exhibit growth retardation, weight loss, and heart defects (Boissel et al., 2009; Christian et al., 2014), whereas *FTO*-overexpressing mice present obesity, weigh gain, and enhanced food intake (Church et al., 2010). Recently, m^6^A sequencing of human T2DM islets revealed that the coefficient of variation of m^6^A methylation regulators is higher in patients with T2DM than in controls, with m^6^A in the patients with T2DM regulating β-cell gene expression to a higher extent than in controls (De Jesus et al., 2019). Further studies have shown that m^6^A methylated RNA and *METTL3* levels are consistently higher in liver tissues of patients with T2DM than in controls; hepatocyte-specific knockout of *METTL3* in mice fed with a high-fat diet (HFD) increases insulin sensitivity and decreases fatty acid synthesis (Xie et al., 2019). *METTL3* significantly modulated HFD-induced metabolic disorders, insulin sensitivity, and hepatogenic diabetes (Li et al., 2020). In addition, m^6^A and RNA sequencing of diabetic hippocampi revealed that the alteration of m^6^A modifications might cause hippocampal neuron damage and further lead to cognitive impairment in patients with T2DM (Song et al., 2020). Collectively, studies have strongly indicated that m^6^A modifications may play a role in the pathogenesis of T2DM, but the biological role and the underlying mechanism of m^6^A still need to be explored.

Co-expression analysis is an effective bioinformatics method that has not been applied to analyze the mechanism of gene regulation. The construction of gene expression regulatory network by co-expression analysis has been widely used in the regulation of growth and development and the pathogenesis of various diseases. However, this method has not been applied to analyze the regulation of m^6^A regulator on T2DM. In the system composed of multiple variables, when studying the influence or correlation degree of one element on another, the influence of other elements is regarded as constant, that is, the influence of other factors is not considered temporarily, and the degree of the relationship between the two elements is studied separately. The numerical results are partial correlation coefficient. Thus, the utilization of partial correlation coefficient to analysis of co-expression is a powerful method. In the reported m^6^A profiling analysis, they only explore the regulatory genes of m^6^A regulator through differential expression (Scuteri et al., 2007; Taneera et al., 2018). Although this analysis method can display more information, it could not reveal the key influencing factors. Co-expression analysis can mine the most critical related genes from a large sample study. If combined with m^6^A profiling, it is relatively more able to dig out the key regulatory mechanisms.

To investigate the correlation of m^6^A modifications with human transcripts expression in patients with T2DM, we collected methylated RNA immunoprecipitation (MeRIP)-seq and RNA-seq transcriptome samples from human islet β-cells, and found 15 differentially expressed m^6^A genes and used these for analysis, including *METTL3*, *FTO*, and *YTHDF1*, etc. We further identified the potential human transcripts associated with m^6^A genes based on the partial correlation coefficient. Our analyses of GO and KEGG pathway enrichment revealed that most of the identified human transcripts were involved in metabolic pathway and MAPK1/MAPK3 signaling, which is a critical biological process controlling the pathogenesis and development of T2DM, suggesting that mRNA m^6^A methylation plays a crucial role in T2DM.

## Materials and Methods

### Datasets

We downloaded the gene expression profile of GSE50398 from the GEO database, a free and openly available database. The dataset used Agilent GPL6244 Platform and includes the genome-wide mRNA expression data of the 178 samples. We downloaded the processed data, that gene and exon expression normalizations have been carried on by the TMM method (Robinson and Oshlack, 2010) and further normalization has been performed using the adjusting the expression to gene or exon length, respectively.

### Partial Correlation

We defined gene expression datasets as random variables *G*, expression of m^6^A regulators (Supplementary Table 1) as random variables *M*, and the expression matrix of diabetes-related genes (Supplementary Table 2) as control variable *D*. The partial correlation coefficient ρ reflects the association between *G* and *M*, where the effects of *D* are removed. Given the linear regression of G with D and the linear regression of M with D, the ρ is calculated as the linear correlation between the two residuals.

The calculation of partial correlation coefficient, the variable m^6^A factor x, the reference variable diabetes factor Z, the variable gene y to be analyzed, and the partial correlation coefficient between x and y were calculated after excluding the influence of Z variable:

$$r_{x\,y.Z}=\frac{r_{x\,y}-r_{x\,Z}\,r_{x\,Z}}{\sqrt{\left(1-r_{x\,Z}^{2}\right)\,\left(1-r_{y\,Z}^{2}\right)}}$$

The H0 of partial correlation coefficient test is the partial correlation coefficient of two variables in the population is 0. Using *t*-test method, the formula is as follows:

$$t=\frac{r\,\sqrt{n-k-2}}{\sqrt{1-r^{2}}}$$

Where r is the corresponding partial correlation coefficient, n is the number of sample observations, K is the number of control variables, and n-k-2 is the degree of freedom. When p < 0.05, the original hypothesis is rejected.

### Differential Expression

We analyzed the differentially-expressed genes in pancreatic islet β-cells between T2DM patients and healthy volunteers using GEO2R, based on R language, an online analysis tool for the GEO database. According to the GEO2R criteria, we identified genes as differentially-expressed if logFC > 2 (upregulated genes) or logFC < -2 (downregulated genes). Significant differences have been statistically tested and the FDR-adjusted P value has been applied, that FDR is an adjusted P value to trim false positive results. We considered adjusted P values < 0.05 as statistically significant and used the calculated value to decrease the false positive rate.

### Gene Ontology and KEGG Pathway Analysis of Differentially Expressed Genes

We used gene ontology (GO) analysis to annotate genes and classify their functions into biological pathways, molecular function, and cellular components (Scuteri et al., 2007). The Kyoto Encyclopedia of Genes and Genomes (KEGG) is a set of databases that disposes biological pathways and genomes related to diseases and drugs. KEGG is a channel promoting an overall and deep understanding of biological systems (Kanehisa, 2002). An FDR-corrected *p*-value has been utilized, and we set a *p* < 0.05 as the cut-off criterion for statistically significant differences. Cellular components, molecular functions, and biological processes were analyzed using the DAVID online database (Huang da et al., 2009a,b).

### Co-expression Network Analysis

We assessed and constructed the co-expression network of differentially-expressed genes. Flat expression patterns across samples in the derivation dataset were filtered out by excluding genes with standard deviations ≤ 0.1. We calculated the partial correlation coefficient, ρ, for each pair of genes, and we defined all gene pairs with ρ ≥ 0.3 as gene-gene associations in the network. In the co-expression network, the nodes represent genes and the edges represent the connections with coefficient ≥ 0.3.

### eQTL Analysis

The trans-eQTL were download from eQTLGen database^1^. This database incorporates 37 datasets with a total of 31,684 individuals and established to identify the downstream consequences of trait-related genetic variants. the cis-eQTL, trans-eQTL, eQTS and replication results are available on this website. This database contains the trans-eQTL results for about 10,000 known genetic risk variants. The statistically significant trans-eQTLs are browsable, the full results were downloaded for further analysis. The m^6^A peaks were defined as a 100 bp region overlapping the m^6^A position which sequenced by Jesus group (De Jesus et al., 2019).

### Software Tools

We used hierarchical cluster analysis to show the volcano plot and heat map of two groups on ImageGP website^2^. The network generation and statistical calculations were analyzed by the (R-platform) Affy package (Gautier et al., 2004). We generated a functional co-expression network annotation using PNATHER (Thomas et al., 2003; Mi et al., 2013). We used the DAVID tool to perform GO enrichment and KEGG analyses, and to estimate network candidate communities on the basis of their associations with functional annotations (Huang da et al., 2009b).

## Results

### Strategy

**FIGURE 1:**
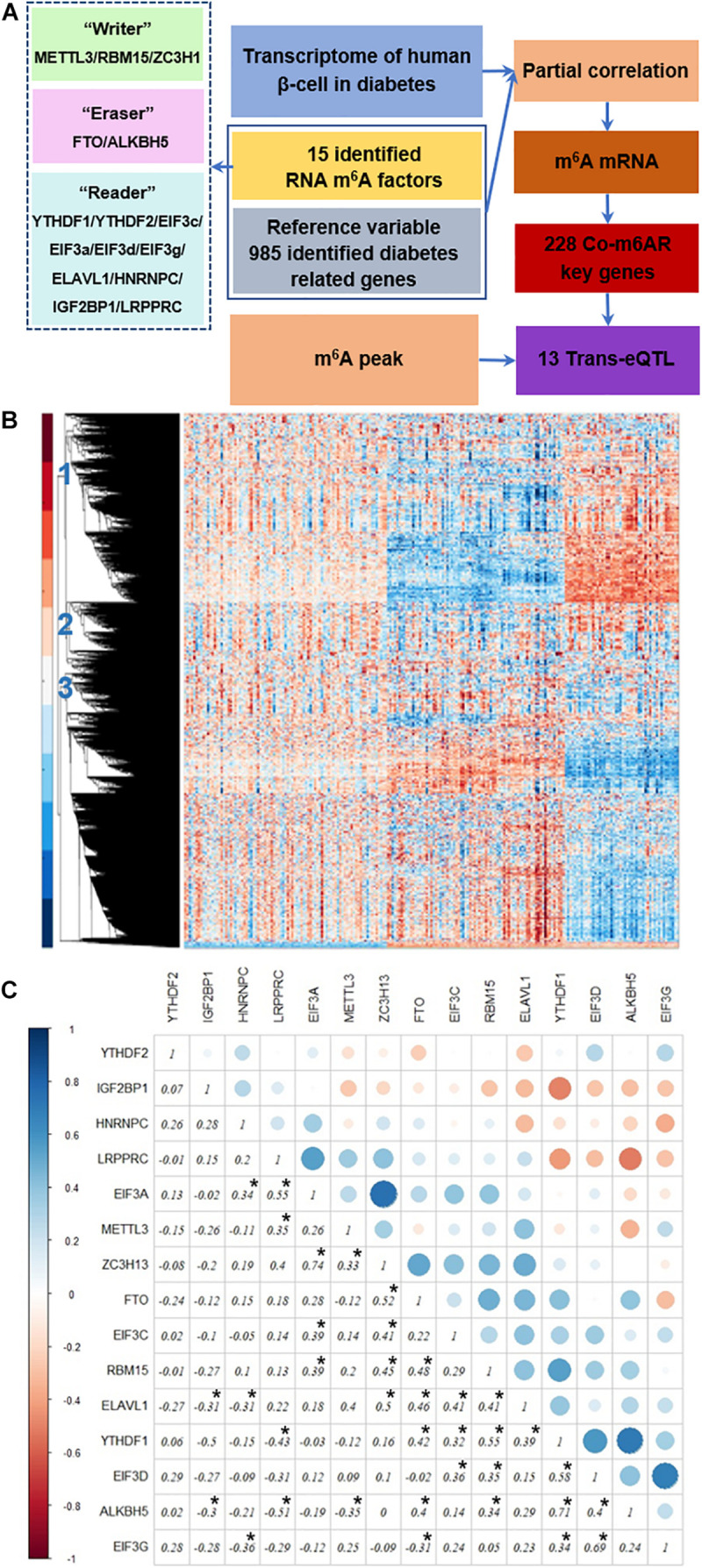
Strategy to identify co-expressed genes with m^6^A regulators. **(A)** Strategy. The transcriptome data of islet β-cells from T2DM patients and controls was applied, then the genes co-expressed with RNA m^6^A regulators was then detected due to the partial correlation coefficient factors, co-m^6^AR genes were further identified based on the published RNA m^6^A modification profiles of β-cells, and finally screened out 13 eQTL-SNPs. **(B)** Transcriptome heatmap of detected genes with their differential expressions in control versus T2DM islet β-cells. Three types of differential expressed genes have been marked. **(C)** Co-expression among m^6^A regulators. The correlation coefficients of 15 RNA m^6^A methylation regulators have been shown and marked with black asterisk.

Figure 1A depicts our analysis strategy. First, we collected the transcriptome data of islet cells from diabetic patients and controls from published datasets. Then, we analyzed the differential expression of RNA m^6^A methylation regulators, including methyltransferases, demethylases, and methylation readers. Then we used genes associated with diabetes pathways as the control variables to calculate partial correlation coefficients, and we screened the genes co-expressed with RNA m^6^A regulators based on partial correlation coefficient factors. Next, we downloaded the published RNA m^6^A modification profiles of islet beta cells, analyzed the overlapping parts with the above identified genes, and further analyzed the potential biological functions of these genes through the GO enrichment and KEGG pathway analysis. Finally, we screened the trans-eQTL localized in the m^6^A peak of genes in our list.

### Co-expression Among RNA m^6^A Regulators in T2DM

We downloaded the transcriptome datasets of human islet beta cells from 178 T2DM cases. These data had been obtained by gene chip detection, and 12261 genes were detected. The transcriptional heatmap in Figure 1B shows detected genes with their differential expressions and classified mainly into three types of expression patterns. We found 15 differentially-expressed genes among 20 RNA m^6^A methylation regulators, including *EIF3C*, *FTO*, *METTL3*, *RBM15*, etc; and 40 differentially-expressed genes among 46 diabetes related factors. We calculated the correlation coefficients of 15 RNA m^6^A methylation regulators by Pearson coefficient, and found a small correlation between them (Figure 1C). Among those, the correlation coefficient between *ZC3H13* and *EIF3a* was 0.74, that between *ALKBH5* and *YTHDF1* was 0.71, that between *EIF3G* and *EIF3D* was 0.69, that between *LRPPRC* and *ALKBH5* was -0.51, that between *LRPPRC* and *YTHDF1* was -0.43, and that between *EIF3G* and *HNRNPC* was −0.36.

### Genes Co-expressed With RNA m^6^A Regulators (Co-m^6^AR)

**FIGURE 2:**
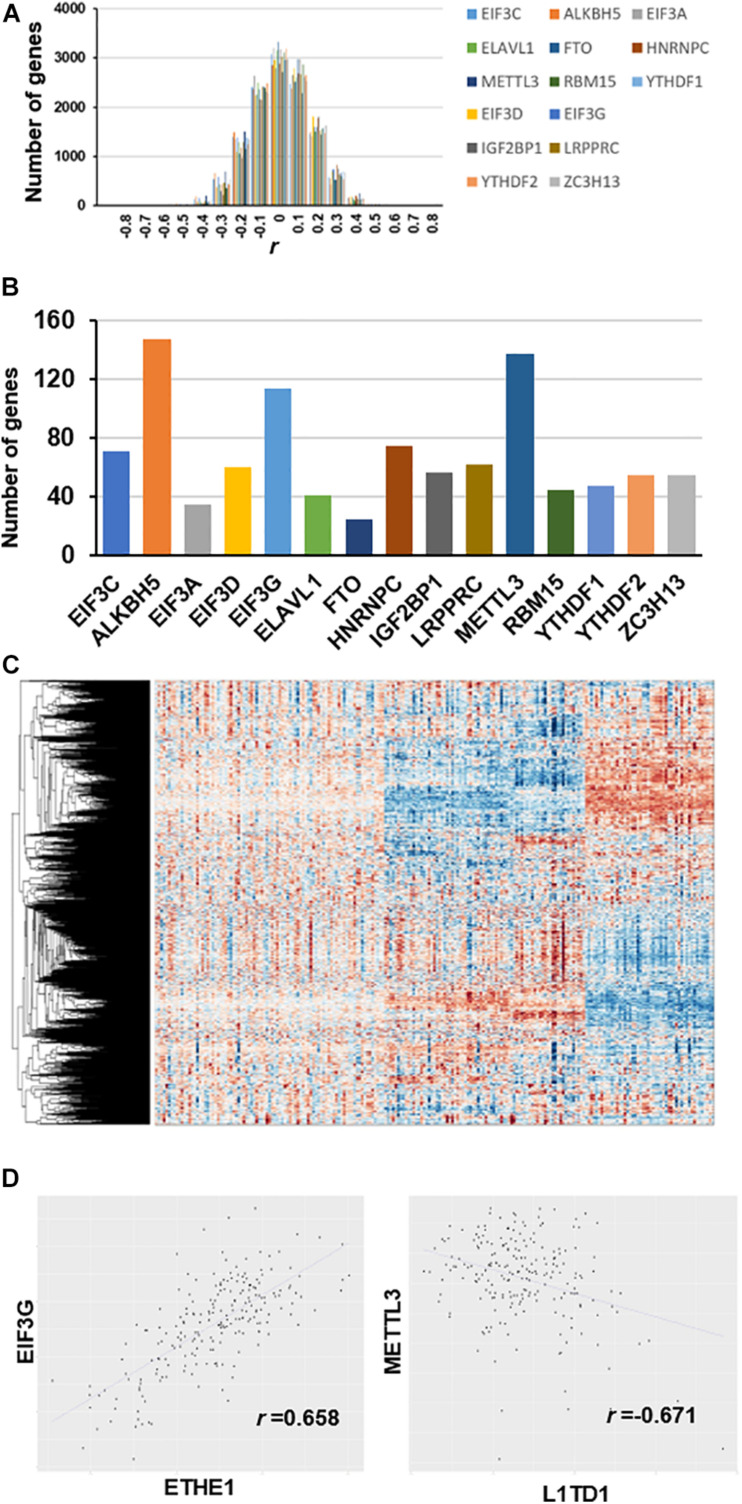
Co-expression between genes and m^6^A regulators after removal of diabetes related gene effects. **(A)** Distribution of partial correlation coefficient. The correlation coefficient distribution ranged from 0.658 to –0.671. **(B)** Number of genes co-expressed with each m^6^A regulator (*r* > 0.35). **(C)** Transcriptome heatmap of differential co-expressed genes in control versus T2DM islet β-cells. **(D)** Scatterplot of gene expression with top value of partial correlation coefficient.

We used partial correlation coefficients to calculate the association between genes and 15 RNA m^6^A regulators (Supplementary Table 3). In the global analysis, the correlation coefficient distribution approximated the normal distribution, ranging from 0.658 to -0.671 (Figure 2A). Figure 2B shows that the maximum correlation coefficient between *EIF3G* and *ETHE1* was 0.658, while the correlation coefficient between *METTL3* and *L1TD1* was the smallest at 0.671. More than 3000 genes had partial correlation coefficients close to 0. We set the threshold value at 0.35; therefore, co-expressed genes have an absolute value of correlation coefficient greater than 0.35. Our results show that the number of genes associated with the expression of *ALKBH5* is the largest (approximately 150) followed by those associated with *METTL3* (approximately 140 genes), and those associated with *EIF3G* expression (approximately 115 genes) (Figure 2C). The number of genes associated with *FTO* expression was the smallest (approximately 30 genes). The heatmap shows the large differences in transcriptional profiles of these clustered genes (Figure 2D).

### Co-m^6^AR Genes Are Enriched in T2DM-Associated Biological Processes

**FIGURE 3:**
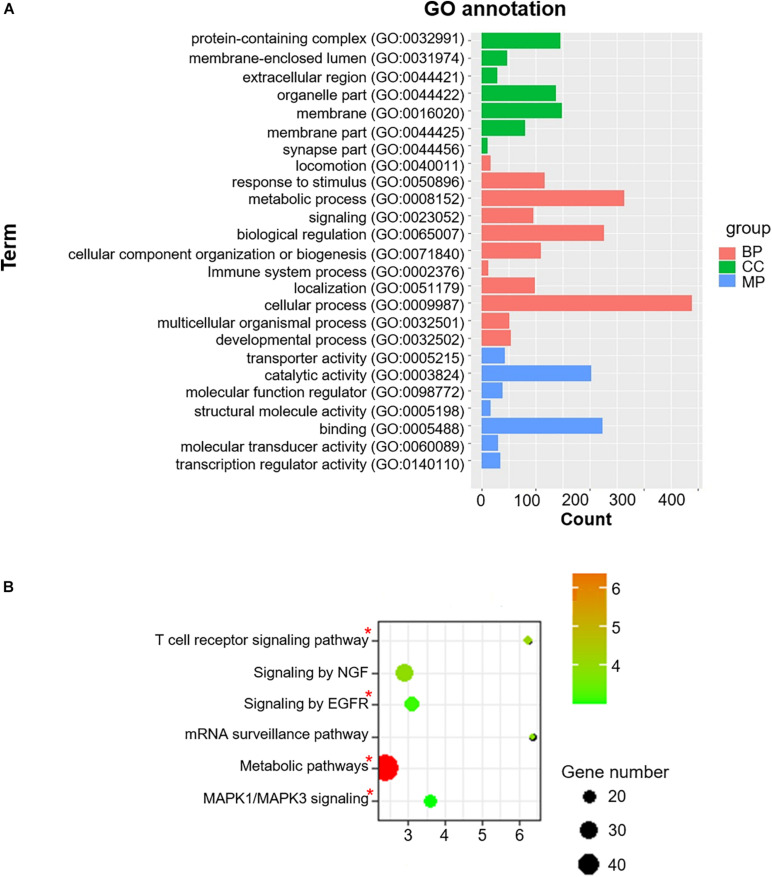
Co-m^6^AR GO functional classification and pathway enrichment analysis. **(A)** Gene-ontology (GO) functional classification. GO analyses of differently expressed genes in control versus T2DM islet β-cells. **(B)** Kyoto Encylopedia of Genes and Genomes (KEGG) pathway enrichment. KEGG analyses of 985 identified m^6^A-associated genes in control versus T2DM islet β-cells. The pathway involved in T2DM development was marked with red asterisk.

To acquire a more comprehensive and deep understanding of the identified differentially-expressed genes, we used the DAVID tool to analyze GO function and KEGG pathway enrichments (Figure 3). The 985 identified m^6^A-associated genes with high significant enrichment levels were enriched in diverse biological processes, such as metabolic process, *MAPK1*/*MAPK3* signaling, the T cell receptor signaling pathway, and *EGFR* signaling (Figure 3B).

### Key Co-m^6^ARs Related to T2DM Revealed by Co-expression Network

We used the co-expression between differentially-expressed genes and the 15 m^6^A methylation factors to generate a network that showed most genes were associated with *METTL3*, *EIF3G*, and *ALKBH5* (Supplementary Figure 1). In Table 1, we show some genes co-expressed with more than one m^6^A regulator. Ankyrin repeat domain 1 (*ANKRD1*) was co-expressed with *ALKBH5* and *METTL3*. Cyclin L2 (*CCNL2*) was co-expressed with *ALKBH5* and *EIF3G*. LIM domain binding 3 (*LDB3*) and Stromal interaction molecule 1 (*STIM1*) were both co-expressed with other m^6^A regulators. Casein Kinase 1 Alpha 1 Like (*CSNK1A1L*) was co-expressed with both *ALKBH* and *METTL3*, which play important role in the hsa04310:Wnt and the hsa04340:Hedgehog signaling pathways. Cysteine sulfinic acid decarboxylase (*CSAD*) was co-expressed with *ALKBH* and *EIF3C* (involved in hsa00430:Taurine and hypotaur metabolism). Cytochrome c oxidase subunit 5A (*COX5A*) was co-expressed with *ALKBH5* and other m^6^A regulators, which play critical roles in hsa00190:Oxidative phosphorylation, hsa01100:Metabolic pathways, hsa04260:Cardiac muscle contraction, hsa04932:Non-alcoholic fatty liver disease (NAFLD), hsa05010:Alzheimer’s disease, hsa05012:Parkinson’s disease, and hsa05016:Huntington’s disease. *GRB2* associated binding protein 2 (*GAB2*) was co-expressed with *METTL3* and *EIF3G* (an important factor in the hsa04014:Ras and hsa04071:Sphingolipid signaling pathways). miR-let-7i was co-expressed with other m^6^A regulators involved in hsa05206: MicroRNAs in cancer.

**TABLE 1:** Typical genes co-expressed with m^6^A regulators in T2DM.

Symbol	*ALKBH5*	*METTL3*	*EIF3G*	*EIF3C*	Others	m^6^A	Diabetes	KEGG_PATHWAY
***GOLGA8B***	Yes	NA	Yes	Yes	NA	NA	NA	
***ANKRD1***	Yes	Yes	NA	NA	NA	NA	Yes	
***BIVM***	Yes	Yes	NA	NA	NA	NA	NA	
***C19orf25***	Yes	Yes	NA	NA	NA	NA	NA	
***RBM26***	Yes	Yes	NA	NA	NA	NA	NA	
***ZFC3H1***	Yes	Yes	NA	NA	NA	NA	NA	
***CSNK1A1L***	Yes	Yes	NA	NA	NA	NA	NA	hsa04310:Wnt signaling pathway, hsa04340:Hedgehog signaling pathway
***AHSA2***	Yes	NA	Yes	NA	NA	NA	NA	
***AP1G2***	Yes	NA	Yes	NA	NA	NA	NA	hsa04142:Lysosome
***ARGLU1***	Yes	NA	Yes	NA	NA	Yes	NA	
***CCNL2***	Yes	NA	Yes	NA	NA	NA	Yes	
***MEG3***	Yes	NA	Yes	NA	NA	Yes	NA	
***PAN2***	Yes	NA	Yes	NA	NA	NA	NA	hsa03018:RNA degradation
***CSAD***	Yes	NA	NA	Yes	NA	NA	Yes	hsa00430:Taurine and hypotaurine metabolism
***COX5A***	Yes	NA	NA	NA	Yes	NA	Yes	hsa00190:Oxidative phosphorylation, hsa04260:Cardiac muscle contraction, hsa05010:Alzheimer’s disease, hsa05012:Parkinson’s disease, hsa05016:Huntington’s disease
***LRRC47***	Yes	NA	NA	NA	Yes	Yes	NA	
***OR10A4***	Yes	NA	NA	NA	Yes	NA	NA	hsa04740:Olfactory transduction
***PRRG2***	Yes	NA	NA	NA	Yes	NA	NA	
***STIP1***	Yes	NA	NA	NA	Yes	Yes	NA	hsa05020:Prion diseases
***EZH1***	NA	Yes	Yes	NA	NA	NA	NA	
***FAU***	NA	Yes	Yes	NA	NA	Yes	Yes	hsa03010:Ribosome
***KIAA0319L***	NA	Yes	Yes	NA	NA	Yes	NA	
***PTGR2***	NA	Yes	Yes	NA	NA	NA	NA	
***SSSCA1***	NA	Yes	Yes	NA	NA	NA	NA	
***KRTAP3-2***	NA	Yes	NA	Yes	NA	NA	NA	
***NUP88***	NA	Yes	NA	Yes	NA	Yes	NA	hsa03013:RNA transport
***CEMIP***	NA	Yes	NA	NA	Yes	NA	NA	
***XAB2***	NA	Yes	NA	NA	Yes	Yes	Yes	hsa03040:Spliceosome
***CRYBB3***	NA	NA	Yes	NA	Yes	NA	NA	
***PTPRH***	NA	NA	Yes	NA	Yes	NA	NA	
***C15orf62***	NA	NA	NA	Yes	Yes	NA	NA	
***FBL***	NA	NA	NA	Yes	Yes	Yes	NA	hsa03008:Ribosome biogenesis
***SPTLC3***	NA	NA	NA	Yes	Yes	NA	NA	hsa00600:Sphingolipid metabolism
***TRIM22***	NA	NA	NA	Yes	Yes	NA	NA	
***MIRLET7I***	NA	NA	NA	NA	Yes	NA	Yes	hsa05206:MicroRNAs in cancer
***C21orf90***	NA	NA	NA	NA	Yes	NA	NA	
***GAB2***	NA	NA	NA	NA	Yes	Yes	Yes	hsa04014:Ras signaling pathway, hsa04071:Sphingolipid signaling pathway
***GADD45GIP1***	NA	NA	NA	NA	Yes	Yes	NA	
***LDB3***	NA	NA	NA	NA	Yes	NA	Yes	
***PID1***	NA	NA	NA	NA	Yes	NA	NA	
***RSF1***	NA	NA	NA	NA	Yes	Yes	NA	
***STIM1***	NA	NA	NA	NA	Yes	NA	Yes	hsa04020:Calcium signaling pathway, hsa04611:Platelet activation
***TEX19***	NA	NA	NA	NA	Yes	NA	NA	

### mRNA of Co-m^6^AR Genes Have m^6^A Methylation in Islet β-Cells

**FIGURE 4:**
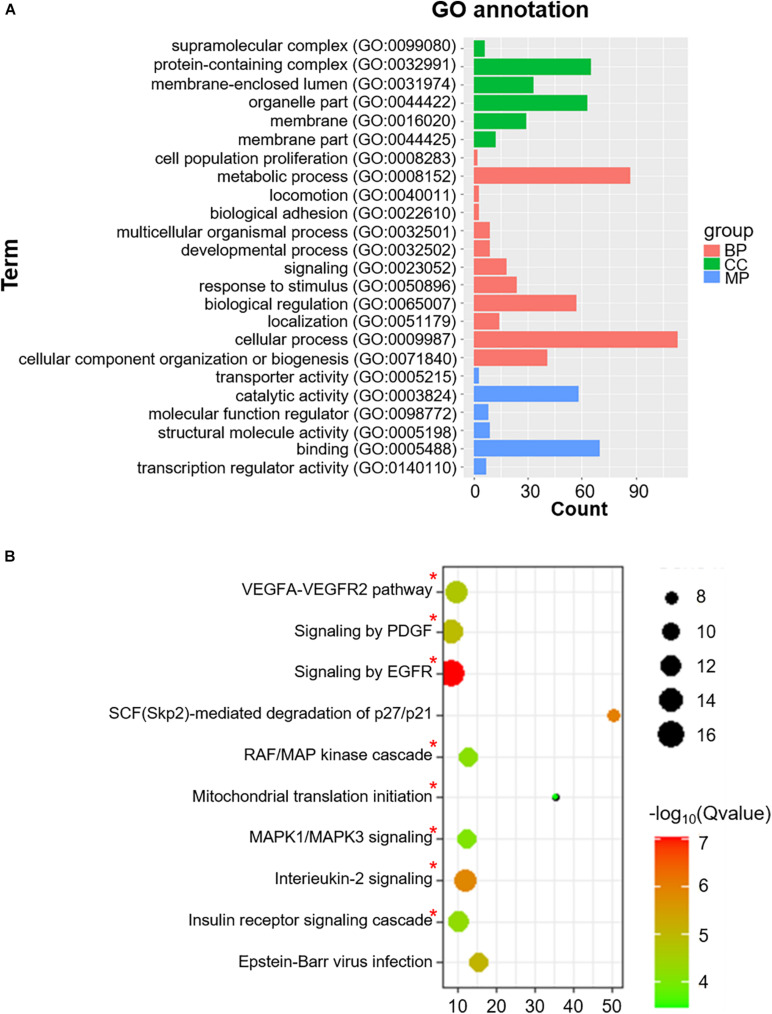
GO functional classification and pathway enrichment analysis of Co-m^6^AR with m^6^A methylation in β-cells. **(A)** GO functional classification. GO analyses of Co-m^6^AR genes in control versus T2DM islet β-cells. **(B)** KEGG pathway enrichment. KEGG analyses of Co-m^6^AR genes in control versus T2DM islet β-cells, and the enrichment pathway involved in T2DM development was marked with red asterisk.

We also identified 228 Co-m^6^ARs with m^6^A methylation based on the MeRIP-seq of human islet β-cells. As shown in Figure 4A, these genes play function in diverse biological processes (BP) such as signaling and stimulus responses. After the enrichment analysis, we found these genes to be enriched in EGFR, interleukin-2, MAPK1/MAPK3, and PDGF signaling, in the signaling cascades of insulin receptor and RAF/MAP kinase, in Epstein-Barr virus infections, and in mitochondrial translation initiation, VEGFA-VEGFR2, and SCF (Skp2)-mediated degradation pathways (Figure 4B).

### Key Co-m^6^ARs With m^6^A Modification in Islet β-Cells Revealed by Co-expression Network

Our co-expression network revealed that most genes were associated with *METTL3*, *EIF3G*, YTHDF1, YTHDF2 and *ALKBH5* (Supplementary Figure 2). In Table 1, we show some genes co-expressed with more than one m^6^A regulator. The gene ubiquitin like and ribosomal protein S30 fusion (*FAU*) was co-expressed with *METTL3* and *EIF3G* (a member of the has03010:Ribosome pathway). XPA binding protein 2 (XAB2) was co-expressed with *METTL3* and other m^6^A regulators involved in the hsa03040:Spliceosome pathway. We mentioned *GRB2* in this network above.

### eQTL Localizaed in the m^6^A Peaks of Genes Co-expressed With m^6^A Regulators

The trans-eQTL were downloaded from eQTLGen database. We identified 13 eQTL-SNP localized in the m^6^A peak region (around 100 bp) which is acquired from the m^6^A-MeRIP-seq of human islet beta cells. In Table 2, there are three eQTL-SNPs localized in chromosome 1, two in chromosome 11, five in chromosome 19. Chromosome 15, 16, and 20 has only 1, respectively. They are belonging to 10 genes. *PHF13*, *SGTA* and *XAB2* contained 2 eQTL-SNP, respectively. Six eQTL-SNPs are involved in “A” allele. Four genes including *GAB2*, *LMNB2*, *XAB2* and *RBM39* are involved in the regulation of diabetes, fat and insulin signaling, according to text mining.

**TABLE 2:** eQTL localized in the m^6^A peaks of genes co-expressed with m^6^A regulators.

SNP	SNP Chr	SNPPos	Assessed allele	Other allele	Gene symbol	Bonferroni P	Reference related to diabetes
rs4908921	1	6613858	T	C	***PHF13***	0.00037375	NA
rs4908922	1	6613888	T	A	***PHF13***	0.00017326	NA
rs16833237	1	151404898	C	T	***POGZ***	9.25E-55	NA
rs7934912	11	78218556	A	T	***GAB2***	9.51E-26	Yes
rs148861080	11	119116390	T	G	***C2CD2L***	2.43E-08	NA
rs78810435	15	73116649	A	G	***NEO1***	8.12E-38	NA
rs138994570	16	57470988	A	G	***POLR2C***	2.43E-06	NA
rs1049910	19	2430637	G	C	***LMNB2***	3.15E-24	Yes
rs7009	19	2754792	A	G	***SGTA***	2.34E-19	NA
rs13282	19	2754810	T	C	***SGTA***	1.79E-05	NA
rs577145	19	7624377	T	C	***XAB2***	2.70E-18	Yes
rs541600	19	7624391	C	T	***XAB2***	3.14E-18	Yes
rs60223674	20	34303255	A	C	***RBM39***	1.11E-27	Yes

## Discussion

Type 2 diabetes mellitus is a polygenic metabolic disease with a pathogenesis in which altered gene expressions at different levels play a crucial role. The analysis of gene expression regulation mechanisms in T2DM is helpful to determine potential therapeutic targets and provide new insights for diabetic therapies. Many m^6^A methylation modifications in eukaryotic mRNA participate in various biological processes by affecting mRNA splicing, translocation, degradation, and translation (Yue et al., 2015; Maity and Das, 2016). Thus, m^6^A can cumulatively regulate the expression of key regulatory genes through multiple effects during the diabetic pathogenic process.

Among the genes corresponding to RNA-seq transcriptomes in pancreatic β-cells, we found 15 m^6^A regulators. Subsequently, we identified the expression of 985 genes significantly correlated with these m^6^A regulators. To investigate the function of these genes, we analyzed their associated pathways. Our GO enrichment analysis assigned the genes to a “metabolic pathway” characterized by metabolic abnormalities, abdominal obesity, hypertension, dyslipidemia, and hyperglycemia. Our results indicate that this pathway is closely associated with T2DM in agreement with studies in which metabolic syndrome is a key etiological factor increasing the risk of T2DM (Hudish et al., 2019; Piche et al., 2020). In addition, other genes were enriched in processes confirmed to be also strongly associated with the pathogenesis and progression of T2DM such as the “MAPK signaling,” “EGFR signaling,” and “T cell receptor signaling pathway.” The findings suggest that m^6^A methylation factors may be modulating the expression of genes in pancreatic β-cells of patients with T2DM.

In addition, we evaluated the above genes based on MeRIP-seq transcriptomes of human islet β-cells, and we further identified the remaining 228 genes co-expressed with m^6^A regulators. We analyzed their associated processes by GO annotations. They were enriched in “epidermal growth factor receptor (EGFR) signaling,” “insulin receptor signaling,” “MAPK1/MAPK3 signaling,” and “RAF/MAP kinase signaling.” Genetic interaction analyses of enhancers and protein-coding genes suggested that EGFR might be a novel susceptibility gene for T2DM (Yang et al., 2020). In the insulin receptor signaling pathway, PIk3R2 is an important regulatory subunit of PI3K/p85, and it can significantly suppress the activation of the PI3K/Akt pathway participating in the physiological and pathological diabetes processes (Cantley, 2002; Taniguchi et al., 2006). In the MAPK signaling pathway, MAPK3 encodes the extracellular signal regulated kinase 1 (*ERK1*), considered a crucial factor for cell proliferation, regulating the insulin gene expression and β-cell survival (Briaud et al., 2003). *ERK1* showed an increased expression in islet β-cells of diabetic mice (Kanda et al., 2009), and *ERK1* knockout mice are significantly resistant to HFD-induced obesity and insulin resistance as compared with control mice (Jager et al., 2011).

Furthermore, we suggest that the genes co-expressed with multiple m^6^A regulators may be critical factors in the regulation of diabetes by m^6^A. Therefore, we classified these genes and analyzed the published data to explore the potential association between these genes and diabetes. Genome-wide association studies revealed SNPs in *CCNL1* and *COX5A* that are significantly associated with the risk of T2DM and a high total urine arsenic content, respectively (Wang et al., 2016; Grau-Perez et al., 2018). Transcriptome studies have found miR-let-7i downregulated in diabetes mice and controlling β-cells (Cheng et al., 2015). The evidence supports that *CSAD* is involvement in a fulminant type of diabetes (Kawabata et al., 2019). The expression of *STIM* expression was low in islets from T2DM pancreas and was associated with proinflammatory cytokines and palmitate (Kono et al., 2018). As we expected, these identified Co-m^6^AR genes are strongly associated with T2DM processes. However, how m^6^A methylation regulates these genes remains unclear.

The m^6^A alterations in mRNA change gene expression profiles and, thus, regulate the pathogenesis and development of various diseases, including tumors, nervous system diseases, and T2DM. We found *EGFR* mRNA to be affected by the expression level of m^6^A methylation factors. This is consistent with previous findings on m^6^A-binding protein *YTHDF2* and modulating the location and stability of *EGFR* mRNA at its 3’UTR site, enhancing the degradation of the *EGFR* mRNA, and playing an anti-tumor role in hepatocellular carcinoma (Yu et al., 2019). *YTHDF2* has also been reported to promote the mRNA expression level of inflammatory response in LPS-stimulated monocyte macrophage cells, such as IL-6, IL-2, IL-1β, and TNF-α(Yu et al., 2019). Another important gene, *ERK*, has also been identified to be regulated by m^6^A, as confirmed by the interaction between *ERK* and *METTL3* and *WTAP*. *METTL3* binds to *USP5* and active ERK-mediates phosphorylation, which makes the m^6^A methyltransferase complex more stable and results in a high level of m^6^A modifications (Prasun, 2020). Taken together, we suggest that the identified genes corresponding to diabetic m^6^A regulators may be crucial for different clinical characteristics of T2DM.

It can be seen that the identified genes are not only co-expressed with m^6^A regulators, but also have m^6^A methylation modifications. Subsequently, we screened these identified genes from the trans-eQTL database, and finally found 13 eQTL-SNPs localized in the m^6^A peak region. Among these eQTL-SNPs, four genes have been reported involved in diabetes. GRB2 associated binding protein 2 (*GAB2*) is an adaptor protein of the insulin receptor substrate 1 family and associated with the downstream signaling from cytokine receptors (Robinson and Oshlack, 2010). XPA binding protein 2 (*XAB2*) exerts as a regulator in hyperglycemia with chronic insulin (Lim et al., 2008). Lamin B2 (*LMNB2*) can regulate fasting blood glucose by increasing insulin secretion or regenerating beta cells (de Toledo et al., 2020). RNA binding motif protein 39 (*RBM39*) were associated with a higher risk of insulin in clinically significant retinopathy of prematurity (Lynch et al., 2016). Obviously, these genes will be of value for studying the prognosis and diagnosis of T2DM, however, the underlying mechanisms of the identified genes in T2DM remain to be elucidated.

In summary, we identified genes co-expressed with m^6^A regulators in human T2DM islets; some of them contained known m^6^A methylation modifications. These genes are enriched in T2DM-related biological processes. These promising genes provide a novel insight into the progression of T2DM and need to be confirmed in further. The identified transcripts may give helpful information for understanding the effects of m^6^A methylation in the prognosis and diagnosis of T2DM.

## Data Availability Statement

The datasets presented in this study can be found in online repositories. The names of the repository/repositories and accession number(s) can be found in the article/Supplementary Material.

## Author Contributions

CC and JT designed the project and wrote the manuscript. SL and MY collected the database. QX and WL did the experiments and analyzed the data. All authors contributed to the article and approved the submitted version.

## Conflict of Interest

The authors declare that the research was conducted in the absence of any commercial or financial relationships that could be construed as a potential conflict of interest.

## Footnotes

1 www.eqtlgen.org

2 http://www.ehbio.com/Cloud_Platform/front/#/

## Supplementary Material

Supplementary Figure 1 Co-m^6^AR co-expression network. Functional protein-protein interaction network of Co-m^6^AR genes in control versus T2DM islet β-cells was shown, the key genes were marked with green asterisk.

Supplementary Figure 2 Co-expression network of Co-m^6^AR with m^6^A methylation in β-cells. Functional protein-protein interaction network of differently expressed genes with *METTL3*, *EIF3G*, *YTHDF1*, *YTHDF2*, and *ALKBH5* was shown, and these five RNA m^6^A methylation regulators were marked with blue asterisk.
