# Supplementary figures and images for: Co-expression Network Revealed Roles of RNA m6A Methylation in Human β-Cell of Type 2 Diabetes Mellitus
Source: Front Cell Dev Biol. 2021 May 18;9:651142. doi: 10.3389/fcell.2021.651142 (PMC8168466; doi:10.3389/fcell.2021.651142)

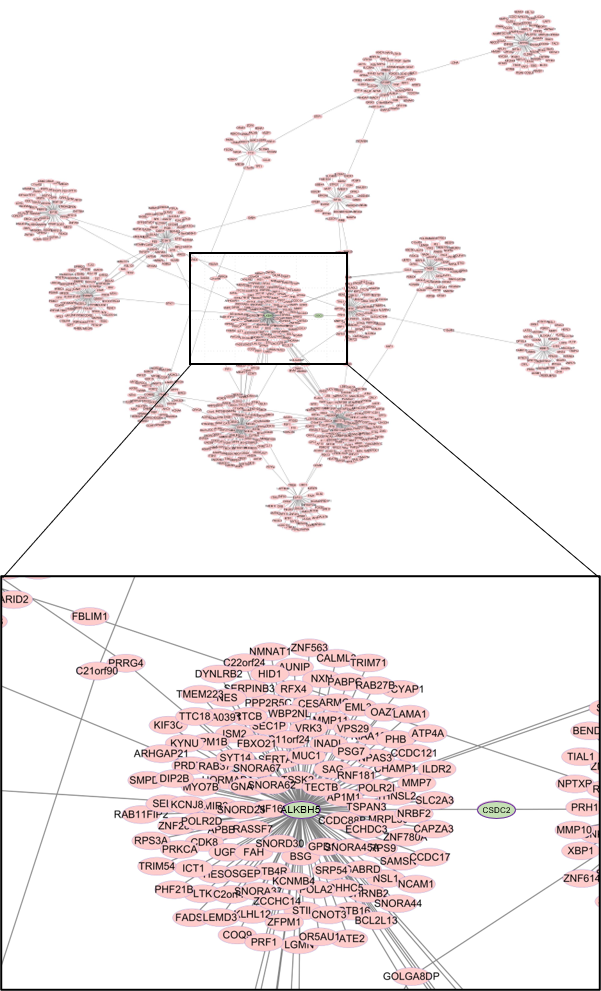

Supplement: Supplementary Figure 1 — Co-m6AR co-expression network. Functional protein-protein interaction network of Co-m6AR genes in control versus T2DM islet β-cells was shown, the key genes were marked with green asterisk. [file Image_1.TIF]

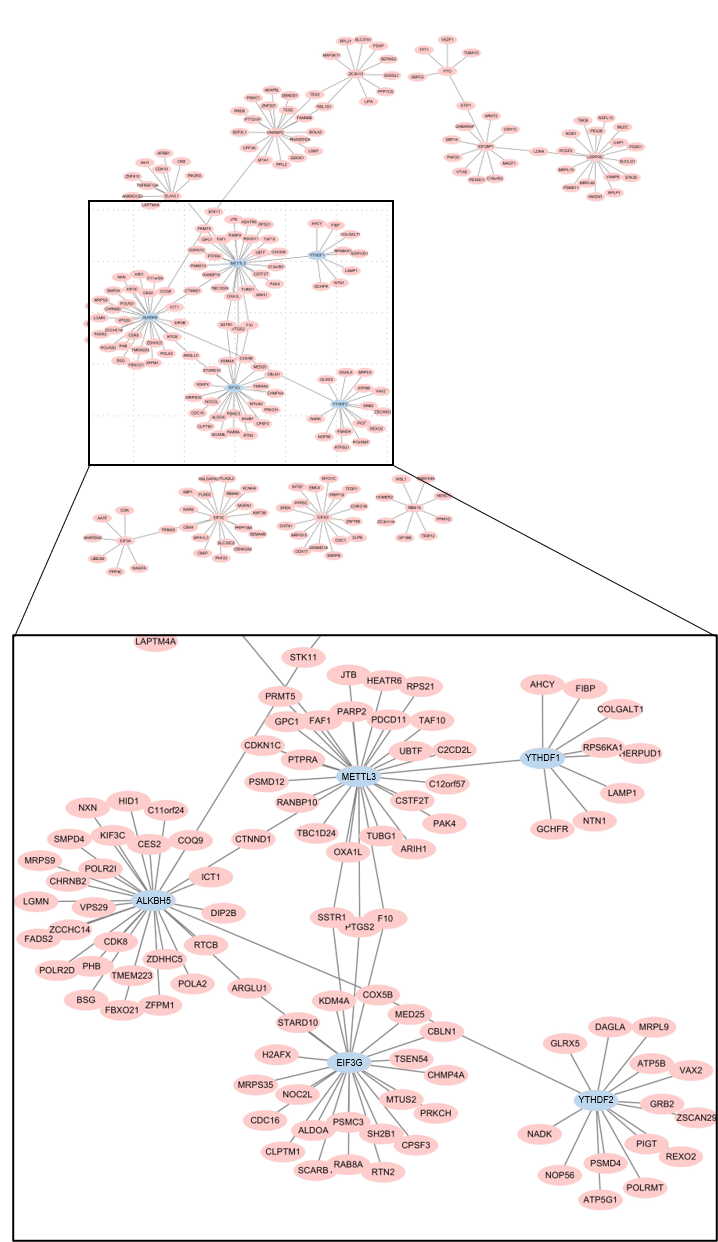

Supplement: Supplementary Figure 2 — Co-expression network of Co-m6AR with m6A methylation in β-cells. Functional protein-protein interaction network of differently expressed genes with METTL3, EIF3G, YTHDF1, YTHDF2, and ALKBH5 was shown, and these five RNA m6A methylation regulators were marked with blue asterisk. [file Image_2.TIF]
